# Preoperative prognostic nutritional index and its impact on surgical-site infection after cesarean section: a retrospective case–control analysis

**DOI:** 10.1007/s00404-026-08376-5

**Published:** 2026-03-05

**Authors:** Karolin Ohanoglu Cetinel, Alperen İnce, Bugra Tunc, Osman Murat Guler, Mustafa Can Sivas, Gorkem Arica

**Affiliations:** 1 Department of Obstetrics and Gynecology, Republic of Turkey Ministry of Health, Basaksehir Cam and Sakura City Hospital, Istanbul, Turkey; 2 Department of Obstetrics and Gynecology, Konya Bozkir State Hospital, Konya, Turkey; 3 Department of Obstetrics and Gynecology, Mersin Aydincik State Hospital, Mersin, Turkey; 4 Department of Obstetrics and Gynecology, Tekirdag Kapakli State Hospital, Tekirdag, Turkey; 5 Cerrahpasa Faculty of Medicine, Department of Surgical Medical Sciences Department of Obstetrics and Gynaecology, Istanbul, Turkey

**Keywords:** Cesarean delivery, C-reactive protein, Prognostic nutritional index (PNI), Surgical-site infection (SSI)

## Abstract

**Purpose:**

Surgical-site infections (SSI) remain a major complication of cesarean delivery, increasing maternal morbidity, hospital stay, and healthcare costs. Although several risk factors have been identified, the role of maternal nutritional status—particularly the Prognostic Nutritional Index (PNI)—has not been well established in obstetric surgery.

**Methods:**

This retrospective case–control study included 190 women who underwent cesarean delivery at a tertiary referral center between 2020 and 2025. Preoperative PNI was calculated using serum albumin and lymphocyte counts obtained within 24 h before surgery. SSI was defined according to the Centers for Disease Control and Prevention criteria. Demographic, perioperative, and postoperative variables were analyzed to evaluate associations between PNI, SSI status, infection severity, and inflammatory markers.

**Results:**

A total of 190 women were analyzed, including 98 cases with surgical-site infection (SSI) and 92 controls without SSI. Although preoperative PNI was not an independent predictor of SSI, lower PNI values were associated with more severe infections requiring broad-spectrum antibiotics, longer hospitalization, and higher postoperative C-reactive protein (CRP) levels. A significant inverse correlation was observed between PNI and CRP among patients with SSI (*r* =  − 0.338, *p* = 0.001). Longer operative duration and smoking were also associated with an increased risk of SSI.

**Conclusion:**

Although PNI was not an independent predictor of SSI, lower values were strongly associated with infection severity and systemic inflammation. Integrating PNI into preoperative risk assessment—alongside body mass index and operative time—may help identify high-risk women. Larger prospective studies are warranted to confirm these findings and to evaluate the impact of nutritional optimization on postoperative outcomes.

## What does this study add to the clinical work

**Table:** 

This study demonstrates that while preoperative PNI is not an independent predictor of surgical-site infection occurrence after cesarean delivery, it is associated with infection severity and systemic inflammatory response. Incorporating nutritional assessment into preoperative evaluation may help identify clinically vulnerable patients and support more individualized postoperative monitoring.	

## Introduction

Surgical-site infection (SSI) is one of the most frequent postoperative complications following cesarean delivery (CD) and is associated with increased maternal morbidity, prolonged hospitalization, unplanned readmissions, and higher healthcare costs [1, 2]. Published literature reports SSI rates after cesarean delivery ranging between approximately 3–10%, depending on case mix, infection control measures, and institutional protocols [1, 2]. Several maternal and perioperative risk factors have been consistently implicated, including elevated body mass index (BMI), prolonged operative time, emergency procedures, chorioamnionitis, and suboptimal wound management [3–5]. Despite standardized antibiotic prophylaxis and improved surgical techniques, SSI remains a substantial clinical and economic burden in obstetric care.

In parallel, increasing attention has been directed toward the impact of perioperative nutritional and inflammatory status on surgical outcomes. Prognostic Nutritional Index (PNI), calculated from serum albumin and total lymphocyte count as originally described by Onodera et al. [6], has emerged as a simple composite marker reflecting both nutritional reserve and cell-mediated immunity. Low PNI has been associated with higher rates of postoperative complications, infectious morbidity, and prolonged hospital stay across various surgical populations, including gastrointestinal, spine, and gynecologic surgery [7–11]. These findings support the concept that impaired immuno-nutritional status predisposes patients to poor wound healing and increased susceptibility to infection.

Evidence specific to obstetric populations is more limited but growing. Studies in women undergoing cesarean section have suggested that hypoalbuminemia and abnormal nutritional or inflammatory markers are associated with delayed wound healing, infectious complications, and adverse peripartum outcomes [12–14]. A multicenter analysis further identified maternal comorbidities and biochemical parameters as contributors to infectious morbidity following CD in high-risk pregnancies [15]. More recently, emerging data have begun to explore the relationship between preoperative nutritional indices and postoperative outcomes in cesarean deliveries, indicating a potential role for immuno-nutritional assessment in this setting [16, 17]. However, robust evidence on the prognostic value of PNI specifically for SSI risk and severity in cesarean patients is still lacking.

Currently, there is no validated PNI threshold specific to obstetric populations. Physiological changes during pregnancy, including hemodilution and immune modulation, alter serum albumin and lymphocyte levels, make the direct application of surgical or oncologic cut-offs inappropriate. In this study, PNI was therefore analyzed as a continuous variable to avoid arbitrary stratification. Future prospective studies are warranted to establish pregnancy-specific reference ranges and clinically meaningful thresholds for obstetric practice.

Given that CD is the most commonly performed major surgical procedure in women worldwide, the identification of objective, inexpensive, and easily obtainable biomarkers to refine SSI risk stratification is of particular clinical importance. PNI, derived from routine preoperative laboratory tests, is a promising candidate for this purpose. Yet, its relevance in the context of cesarean delivery and post-cesarean SSI has not been clearly established.

Therefore, the present study aimed to investigate the association between the preoperative PNI and the occurrence and severity of SSI following cesarean section in a well-defined cohort from a tertiary referral center. We hypothesized that lower preoperative PNI would be associated not only with the presence of SSI but also with more severe infectious courses and heightened systemic inflammatory response.

## Methods

### Study design and setting

This retrospective case–control study was conducted at the Department of Obstetrics and Gynecology of Başakşehir Çam and Sakura City Hospital, a fourth-level tertiary referral center in Istanbul, Turkey, between May 2020 and March 2025. The study protocol was approved by the institutional ethics committee (approval no. 2025-91 date: 24-03-2025) and performed in accordance with the Declaration of Helsinki. This study was designed as a comparative analysis to evaluate the association between preoperative Prognostic Nutritional Index (PNI) and the presence and severity of surgical-site infection (SSI), rather than to estimate SSI incidence.

### Patient selection and eligibility

All consecutive women who underwent cesarean delivery during the study period were screened for eligibility. A total of 1.240 cesarean deliveries were initially evaluated.

Inclusion criteria were:

1. Availability of preoperative serum albumin and complete blood count results obtained within 24 h before surgery, and postoperative day 1 C-reactive protein (CRP) measurements;
2. Maternal age between 18 and 35 years;
3. Women with a history of previous cesarean delivery, or those scheduled for an elective cesarean section in the current pregnancy due to obstetric indications such as breech or transverse fetal presentation, cephalopelvic disproportion, multiple pregnancy, or suspected fetal macrosomia;
4. Patients admitted for routine antenatal follow-up whose non-stress test (NST) unexpectedly demonstrated fetal distress leading to cesarean delivery.

Exclusion criteria included:

1. Patients initially admitted for vaginal delivery who subsequently required cesarean section during labor;
2. Any known chronic systemic or autoimmune disease (such as diabetes mellitus, gestational diabetes, renal or hepatic disease, or autoimmune disorders etc.);
3. Any documented comorbidity that could affect postoperative healing, including hypothyroidism, cardiovascular disease, or other chronic medical conditions;
4. Patients with placental abnormalities, such as placenta previa, placental abruption (abruptio placentae), or other placental pathologies;
5. Patients with amniotic fluid disorders including anhydramnios or polyhydramnios;
6. Preexisting infection or antibiotic use before delivery;
7. Missing preoperative laboratory data; and
8. Inadequate postoperative follow-up.

The hospital is a national tertiary referral center for high-risk pregnancies. Therefore, women with complex obstetric comorbidities or under perinatology follow-up were intentionally excluded to minimize confounding and to ensure sample homogeneity.

After applying these criteria, 190 women were found eligible and included in the final analysis. Accordingly, the final analytical sample was intentionally constructed to include women with SSI and an eligible non-SSI comparison group drawn from the same source population. The main reasons for exclusion were missing laboratory results, medical comorbidities, and high-risk obstetric conditions such as placental or amniotic fluid abnormalities.

### Surgical procedure and postoperative care

All cesarean deliveries were performed by experienced obstetricians using a low-transverse uterine incision. The uterine, fascial, and subcutaneous layers were routinely closed, and the skin was approximated with absorbable subcuticular sutures. Prophylactic antibiotics (2 g cefazolin IV) were administered within 60 min before skin incision for elective cases and as soon as possible after the decision for surgery in emergency cesarean deliveries. Patients remained hospitalized for at least 48 h postoperatively, and daily wound inspections were performed. Elective cesarean deliveries included patients scheduled in advance due to obstetric indications, whereas emergency procedures were performed in women whose routine antenatal follow-up revealed non-reassuring fetal heart rate patterns or fetal distress findings on non-stress testing (NST).

Patients without evidence of severe wound infection or systemic involvement routinely received a combination of cefazolin and metronidazole as standard prophylaxis and postoperative therapy. In cases of resistant or complicated infections, broad-spectrum antibiotics such as piperacillin–tazobactam (Tazocin), meropenem, or teicoplanin were initiated in consultation with the Infectious Diseases Department, based on clinical severity and wound culture results. SSI was defined and classified as superficial, deep, or organ/space according to the National Healthcare Safety Network (NHSN) and Centers for Disease Control and Prevention (CDC) criteria within 30 days of surgery [18].

### Data collection

Demographic and clinical data were retrieved from the hospital’s electronic medical record system, including maternal age, BMI, gravidity, parity, smoking status, operative time, and length of hospital stay. Laboratory variables included preoperative hemoglobin, serum albumin, lymphocyte, and white blood cell counts, as well as postoperative day 1 CRP levels.

The Prognostic Nutritional Index (PNI) was calculated using the following formula, as originally defined by Onodera et al*.* [6]:

$${\mathrm{PNI}} = \left( {10 \times {\mathrm{albumin}}\left[ {{\mathrm{g}}/{\mathrm{dL}}} \right]} \right) + \left( {0.005 \times {\mathrm{lympocyte}}\,{\mathrm{count}}\left[ {/{\mathrm{mm}}^{3} } \right]} \right)$$

### Statistical analysis

Normality of continuous variables was assessed using the Shapiro–Wilk test. Data were summarized as mean ± standard deviation (SD) or median (interquartile range, IQR), as appropriate. Group comparisons were performed using the independent-samples *t *test or Mann–Whitney *U* test for continuous variables and the χ^2^ test or Fisher’s exact test for categorical variables. Given the absence of validated obstetric PNI thresholds and physiological pregnancy-related changes in albumin and lymphocyte counts, PNI was analyzed as a continuous variable rather than dichotomized using a cut-off.

Correlations between PNI and C-reactive protein (CRP) were assessed using Spearman’s rho.

Variables with a *p* value < 0.10 in univariate analysis were entered into a multivariate logistic regression model to identify independent predictors of SSI. Results were expressed as odds ratios (OR) with 95% confidence intervals (CI). A *p* value < 0.05 was considered statistically significant. All analyses were performed using SPSS version 22.0 (IBM Corp., Armonk, NY, USA) or later.

## Results

The present study comprised two comparison groups: individuals diagnosed with surgical-site infection (SSI; *n* = 98) and a control group without SSI (non-SSI; *n* = 92). Comparative statistical analysis was performed to evaluate demographic characteristics, clinical parameters, and perioperative variables between groups.

BMI values were significantly higher in the SSI group (median = 31.6) compared with the non-SSI group (median = 30.09; *p* = 0.031). However, there were no significant differences between groups in age, gravidity, parity, preoperative hemoglobin (Hb), lymphocyte count, albumin, or PNI values (all *p* > 0.05). WBC counts were significantly higher in the non-SSI group (median = 13.57) than in the SSI group (median = 11.33; *p* < 0.001) (Table 1).

**Table 1:** Comparison of Age, BMI, Gravidity, Parity, Hb, WBC, Lymphocyte, Albumin, and PNI Values Between Groups

	Group	N	Median	Min	Max	Z	*p* value
Age	SSI	98	29	18	35	−0.103	0.918
	non-SSI	92	29	18	35		
BMI	SSI	98	31.6	19.81	52.34	−2.157	0.031
	non-SSI	92	30.09	19.71	39.33		
Gravidity	SSI	98	2	1	9	−0.240	0.811
	non-SSI	92	2	1	6		
Parity	SSI	98	0	0	5	−0.050	0.960
	non-SSI	92	0	0	5		
Hb	SSI	98	10.7	7.9	13.2	−0.452	0.652
	non-SSI	92	10.75	7.2	13.6		
WBC	SSI	98	11.33	4.77	27.69	−3.816	< 0.001
	non-SSI	92	13.57	6.88	19.94		
Lymphocyte	SSI	98	1.73	0.42	4.31	−0.189	0.85
	non-SSI	92	1.75	0.52	3.1		
Albumin	SSI	98	3.6	2.1	5.0	−1.389	0.165
	non-SSI	92	3.4	2.2	4.9		
PNI	SSI	98	44.2	26.8	63.9	−0.865	0.387
	non-SSI	92	43.43	27	57.6		

BMI, Body Mass Index; Hb, Hemoglobin; WBC, White Blood Cell; SSI, Surgical-Site Infection

*p* < 0.05; Mann–Whitney U Test

A significant difference was observed in smoking status (*p* < 0.05), with smokers being more frequent among SSI cases, whereas the frequency of multiple pregnancy did not differ significantly between the groups (*p* > 0.05) (Table 2).

**Table 2:** Comparison of Smoking Status and Multiple Pregnancy Between SSI and Control Groups

			Group		*p* value
			SSI	non-SSI	
Smoking Status^a^	No	n	87	91	0.004
		%	88.8%	98.9%	
	Yes	n	11	1	
		%	11.2%	1.1%	
Multiple Pregnancy^b^	No	n	96	87	0.267
		%	98.0%	94.6%	
	Yes	n	2	5	
		%	2.0%	5.4%	

*p* < 0.05

^a^Pearson’s Chi-Square Test

^b^Fisher's Exact Chi-Square Test

Perioperative characteristics were comparable between groups with respect to type of cesarean delivery (emergency vs. elective), type of anesthesia, and postoperative transfusion requirement (all *p* > 0.05). Drain use was significantly higher in the non-SSI group (p < 0.001) (Table 3).

**Table 3:** Comparison of Cesarean Type, Anesthesia Type, Postoperative Transfusion Requirement, and Drain Use Between Groups

			Group		*p* value
			SSI	non-SSI	
Type of CD^a^	Emergency	n	29	22	0.377
		%	29.6%	23.9%	
	Elective	n	69	70	
		%	70.4%	76.1%	
Type of Anesthesia^b^	Spinal	n	79	76	0.111
		%	80.6%	82.6%	
	General	n	19	12	
		%	19.4%	13.0%	
	Spinal + General	n	0	3	
		%	0.0%	3.3%	
	Spinal + Epidural	n	0	1	
		%	0.0%	1.1%	
Postoperative blood transfusion^c^	No	n	85	85	0.422
		%	86.7%	92.4%	
	Yes(1U)	n	3	3	
		%	3.1%	3.3%	
	Yes (2U)	n	9	4	
		%	9.2%	4.3%	
	Yes (3U)	n	1	0	
		%	1.0%	0.0%	
Drain	No	n	97	78	< 0.001
		%	99.0%	84.8%	
	Yes	n	1	14	
		%	1.0%	15.2%	

*p* < *0.05*

^a^Pearson’s Chi-Square Test; CD: Cesarean Delivery

^b^Fisher’s Exact Test

^c^Postoperative blood transfusion: Yes (1U), Yes (2U), and Yes (3U) indicate the number of blood transfusion units administered postoperatively

Hospitalization was significantly longer in the SSI group (median 8 days) compared with the non-SSI group (median 4 days; *p* < 0.001). Similarly, operative time was significantly longer among SSI cases (median 35 min) than non-SSI cases (median 29 min; *p* = 0.014) (Table 4).

**Table 4:** Comparison of Cesarean Section Duration and Hospital Stay Duration Between Groups

	Group	n	Median	Min	Max	Z	*p* value
Length of Hospital Stay (days)	SSI	98	8.00	3.00	35	-9.784	< 0.001
	non-SSI	92	4.00	2.00	9		
C/S duration (minutes)	SSI	98	35.00	22.00	120	-2.47	0.014
	non-SSI	92	29.00	13.00	97		

*p* < 0.05; Mann–Whitney U Test

Within the SSI cohort, patients receiving antibiotic therapy with Tazocin, Meropenem, or Teicoplanin had significantly lower PNI values (mean = 42.21 ± 7.65) than those treated with Cefazolin or Metronidazole (mean = 46.59 ± 7.20; *p* = 0.031). Hospital stay was also longer among patients receiving broad-spectrum antibiotics (median = 8.5 days) compared with those receiving standard regimens (median = 7 days; *p* = 0.003) (Table 5).

**Table 5:** Comparison of PNI Values and Hospital Stay Duration Based on Antibiotic Regimen in the SSI Group

Antibiotic Regimen	n	PNI Mean ± SD	Hospital Stay Median (Min–Max)	PNI t	PNI *p* value	Stay Z	Stay *p *value
Tazocin, Meropenem, Teicoplanin	18^a^	42.21 ± 7.65	8.5 (6–16)	-2.205	0.031	−2.933	0.003
Cefazolin, Metronidazole	55^a^	46.59 ± 7.20	7 (3–15)	–	–	–	–

PNI, Prognostic Nutritional Index; SSI, Surgical-Site Infection

Independent-samples *t* test was used for comparison of mean PNI values; Mann–Whitney U test was used for comparison of hospital stay durations

*p* < 0.05 was considered statistically significant

^a^Antibiotic regimen data were available for 73 patients in the SSI group; patients with missing data or alternative regimens were excluded from this analysis

A significant negative correlation was found between PNI and postoperative C-reactive protein (CRP) levels within the SSI group (*r* = –0.338, p = 0.001) (Table 6).

**Table 6:** Analysis of the Relationship Between PNI Values and CRP Levels Among Participants in the SSI Group

R		*p *value
PNI vs. CRP	−0.338^a^	0 .001

PNI: Prognostic Nutritional Index; CRP: C-reactive protein; SSI: Surgical-site infection

*p* < 0.01 (two-tailed) was considered statistically significant (r significant at the 0.01 level)

^a^Spearman’s correlation analysis

Multivariate logistic regression analysis identified higher body mass index, longer cesarean duration, and smoking as independent risk factors for surgical-site infection. Specifically, higher BMI (OR 1.07, 95% CI 1.01–1.14; *p* = 0.033), longer operative time (OR 1.02 per minute, 95% CI 1.00–1.04; *p* = 0.028), and smoking (OR 12.85, 95% CI 1.59–104.20; p = 0.017) remained significantly associated with SSI after adjustment. In contrast, age, preoperative PNI, and emergency cesarean delivery were not independently associated with SSI (Table 7).

**Table 7:** Multivariate Logistic Regression Analysis of Risk Factors Associated With Surgical-Site Infection (SSI)

Variable	Adjusted OR	95% CI	*p *value
Age (years)	0.99	0.94–1.05	0.815
PNI (per unit increase)	1.04	1.00–1.09	0.080
BMI (kg/m^2^, per unit increase)	1.07	1.01–1.14	0.033
C/S duration (per minute)	1.02	1.00–1.04	0.028
Emergency C/S (vs. elective)	1.60	0.79–3.22	0.188
Smoker (yes vs. no)	12.85	1.59–104.20	0.017

OR, odds ratio; CI, confidence interval; PNI, Prognostic Nutritional Index; C/S, cesarean section; SSI, surgical-site infection

Multivariate logistic regression analysis including variables with *p* < 0.10 in univariate analysis

## Discussion

The primary objective of this study was to evaluate the predictive utility of the preoperative Prognostic Nutritional Index (PNI) for identifying the risk of surgical-site infection (SSI) following cesarean delivery. Although PNI was not an independent predictor of SSI in multivariate analysis, subgroup findings suggested that impaired nutritional and immune status may contribute to more severe postoperative courses.

Consistent with previous literature, elevated BMI was identified as a significant risk factor for SSI in the present cohort. Olsen et al. demonstrated BMI to be an independent predictor of post-cesarean SSI (adjusted OR 1.1 per unit increase) [3], while Ousey et al. reported BMI as the only significant predictor of SSI in emergency cesarean deliveries (OR 1.17) [4]. Similar findings were described by Meijs et al., who noted that increasing BMI was associated with higher SSI rates due to impaired tissue oxygenation, reduced antibiotic penetration, and prolonged operative time [5]. In our multivariate model, both higher BMI and longer operative duration independently predicted SSI, reaffirming the link between adiposity, surgical complexity, and infection risk.

Smoking also emerged as an independent predictor of SSI. Although our analysis did not assess cumulative exposure, the biological plausibility is well established—nicotine impairs microvascular perfusion, collagen synthesis, and tissue oxygenation, delaying wound healing. These results echo findings from Wyatt et al., who emphasized the role of standardized closure techniques and perioperative counseling to mitigate wound complications after cesarean delivery [19].

A notable observation in our study was the relationship between lower PNI values and more severe postoperative infection. Patients with lower PNI values required broader-spectrum antibiotics and had longer hospital stays, reflecting a more complicated postoperative course. While PNI did not demonstrate strong discriminatory power for SSI occurrence, it was associated with indicators of greater infection burden and slower recovery. Ikeguchi et al. similarly reported that a low preoperative PNI independently predicted SSI after colorectal surgery [12], while Ushirozako et al. and Xi et al. showed comparable associations in spine and gastric surgery cohorts [10, 11]. However, in obstetric populations, this relationship appears to be indirect—reflecting the overall immuno-nutritional reserve rather than directly influencing infection risk.

Importantly, this association should be interpreted with caution. The link between low PNI and the use of broad-spectrum antibiotics likely reflects clinical decision-making rather than a direct biological effect. Patients perceived as more severely ill or exhibiting delayed wound healing may have prompted escalation of antibiotic therapy, indicating that PNI reflects general immuno-nutritional status rather than determining antibiotic choice.

While overall PNI and CRP values did not differ significantly between the SSI and control groups, a significant negative correlation was observed between PNI and CRP within the SSI subgroup (r = –0.338, *p* = 0.001). The higher WBC levels observed in the non-SSI group may reflect physiological peripartum leukocytosis or differences in the timing of blood sampling rather than a protective effect against infection.

This finding suggests that lower preoperative PNI values were associated with higher postoperative CRP levels, consistent with a more intense inflammatory response and greater infection severity. Similar relationships have been reported in gastrointestinal, oncologic, and gynecologic surgery, where low PNI and elevated CRP have been linked to postoperative infection, delayed healing, and prolonged hospitalization [10–13, 15, 16]. In our multivariate model, preoperative PNI did not independently predict SSI occurrence, whereas BMI, smoking, and operative time remained significant risk factors, supporting the notion that PNI may be more closely related to infection severity than to infection occurrence. Similarly, in obstetric populations, Zhou et al*.* demonstrated that women with hypoalbuminemia and heightened inflammatory responses were at greater risk of wound complications after cesarean section [15].

From a clinical standpoint, incorporating nutritional and inflammatory assessment into preoperative evaluation may enhance risk stratification. Although our findings do not establish causality, they support the importance of identifying nutritionally vulnerable patients before surgery. Routine measurement of PNI, in combination with established clinical factors such as BMI, smoking status, and operative duration, may inform perioperative optimization and targeted surveillance strategies in cesarean populations.

Overall, our findings highlight the interconnected roles of nutritional status, obesity, smoking, and surgical characteristics as determinants of SSI after cesarean delivery. These results align with international data reporting post-cesarean SSI rates of 3–15% [1, 2, 11] and reinforce the potential value of PNI as an inexpensive adjunct to conventional risk assessment. Further prospective, multicenter studies are warranted to validate these associations and to explore whether perioperative nutritional optimization can reduce SSI-related maternal morbidity and healthcare costs.

## Limitations

- This study has several limitations. Its retrospective, single-center design may limit generalizability and is subject to selection bias. Strict exclusion of women with significant comorbidities, incomplete laboratory data, or high-risk obstetric conditions reduced the sample size but improved cohort homogeneity. Residual confounding from unmeasured variables, such as micronutrient status or perioperative glycemic control, cannot be excluded.
- Although preoperative Prognostic Nutritional Index (PNI) was evaluated as a continuous variable, no validated obstetric thresholds currently exist, and physiological pregnancy-related changes in albumin and lymphocyte counts may influence absolute PNI values. In line with these considerations, PNI was not independently associated with SSI occurrence in multivariate analysis, suggesting that it may be more informative for assessing infection severity and inflammatory burden rather than serving as a standalone marker.

## Conclusion

Preoperative PNI may serve as a practical marker of postoperative infection severity. However, BMI, operative duration, and smoking were the main independent predictors of SSI. Incorporating PNI into preoperative assessment, together with these factors, may improve risk stratification. Prospective studies are warranted to confirm its prognostic value and potential for guiding nutritional interventions [7–9, 12–16, 19].

## Data Availability

All relevant data are within the paper and can be provided by corresponding author if necessary.
